# Unlocking Arene Phosphorescence in Bismuth–Organic Materials

**DOI:** 10.1021/acs.inorgchem.4c00606

**Published:** 2024-06-01

**Authors:** Alexander C. Marwitz, Anuj K. Dutta, Robin L. Conner, Lulio A. Sanz, Luiz G. Jacobsohn, Karah E. Knope

**Affiliations:** † Department of Chemistry, Georgetown University, Washington, District of Columbia 20057, United States of America; ‡ Department of Materials Science and Engineering, Clemson University, Clemson, South Carolina 29634, United States of America

## Abstract

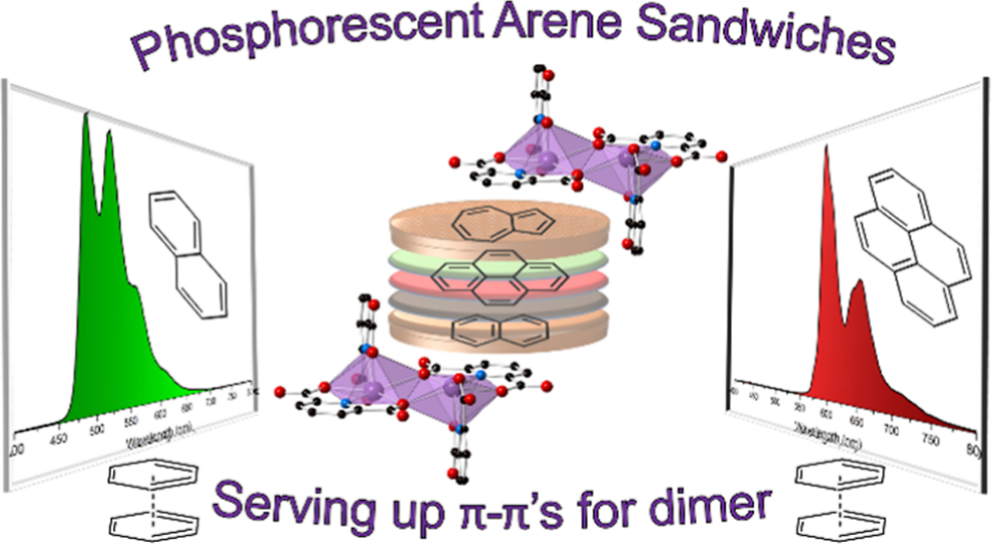

Three novel bismuth–organic compounds, with the general formula [Bi_2_(HPDC)_2_(PDC)_2_]·(arene)·2H_2_O (H_2_PDC = 2,6-pyridinedicarboxylic acid; arene = pyrene, naphthalene, and azulene), that consist of neutral dinuclear Bi-pyridinedicarboxylate complexes and outer coordination sphere arene molecules were synthesized and structurally characterized. The structures of all three phases exhibit strong π–π stacking interactions between the Bi-bound PDC/HPDC and outer sphere organic molecules; these interactions effectively sandwich the arene molecules between bismuth complexes and thereby prevent molecular vibrations. Upon UV irradiation, the compounds containing pyrene and naphthalene displayed red and green emission, respectively, with quantum yields of 1.3(2) and 30.8(4)%. The emission was found to originate from the T_1_ → S_0_ transition of the corresponding arene and result in phosphorescence characteristic of the arene employed. By comparison, the azulene-containing compound displayed very weak blue-purple phosphorescence of unknown origin and is a rare example of T_2_ → S_0_ emission from azulene. The pyrene- and naphthalene-containing compounds both display radioluminescence, with intensities of 11 and 38% relative to bismuth germanate, respectively. Collectively, these results provide further insights into the structure–property relationships that underpin luminescence from Bi-based materials and highlight the utility of Bi–organic molecules in the realization of organic emission.

## Abstract 2

Bismuth compounds containing arene molecules “sandwiched” in the outer coordination sphere via π−π interactions displayed phosphorescence and radioluminescence characteristic of the arene molecule. Previous studies highlighted the importance of the π−π interactions, while this study emphasizes the relative unimportance of hydrogen bonding toward achieving phosphorescence.

## Introduction

Luminescent materials are vital to life in the 21st century and such recognition has led to significant innovations in the design of materials for use in electronic displays^1−8^ and sensors.^9−13^ With respect to the latter, there exist a wide range of analytes of interest for sensing applications. Of particular note are recent advances in the detection of toxic or carcinogenic chemicals such as polycyclic aromatic hydrocarbons (PAHs).^14−16^ While every application that employs luminescent compounds has specific criteria, materials with tunable luminescent properties that are afforded via simple compositional changes are particularly attractive as they can be used as multifunctional materials to meet the demands of a wide range of applications. For example, lanthanide-based phosphors have been synthesized that can act as white-light-emitting diodes, sensors, or anticounterfeiting tags by altering the composition of the material.^17−19^ However, in addition to functionality, the cost and toxicity of the structural components must be kept in mind.

Bismuth has become an attractive element toward the design of functional materials due to its nontoxicity as well as its unique electronic and structural properties.^20−27^ For example, Vogler and colleagues proposed that bismuth and other main group metals with closed-shell *n*s^2^ electron configurations could undergo similar photoluminescent transitions to those observed for the d^10^ metals.^28,29^ In fact, more recent work has shown that bismuth–organic compounds have even more versatility as luminescent materials than originally hypothesized; bismuth has been shown to participate in the frontier orbital electronic transitions directly or indirectly by promoting intraligand transitions through its unique coordination chemistry. Notably, bismuth-based metal-to-ligand charge-transfer (MLCT) materials in which the metal directly participates in the electronic transitions have displayed emission across the visible spectrum and exhibited other attractive properties such as mechanochromic luminescence.^30−33^ In 2021, for example, Marshak et al. reported a bismuth tris(benzo[*h*]quinoline) (bzq) compound, Bi(bzq)_3_, that displayed ^3^MLCT emission in the blue region.^34^ The electronic structure of this compound was found to be similar to a lead perovskite, methylammonium lead iodide, and the composition of the material was analogous to Ir(bzq)_3_, a phosphorescent emitter of interest for organic light-emitting diodes.^35,36^ In a related vein, bismuth halides have shown a propensity to display halide-metal-to-ligand charge transfer (XMLCT) that allows for even more diverse emissive pathways and properties.^37−39^ Further electronic participation from bismuth toward photoluminescence can arise from its metal-based transitions, particularly the ^3^P_1_ → ^1^S_0_ transition and the forbidden ^3^P_0_ → ^1^S_0_ transition.^40^

Bismuth-based materials have also been shown to act as structural scaffolds or hosts to achieve photoluminescence originating from an organic fluorophore. In 2010 and 2011, zur Loye and colleagues reported a series of Bi-2,5-pyridinedicarboxylate coordination polymers that displayed intraligand transitions and yielded blue, green, and white light emission.^41−43^ Additionally, bismuth–organic materials have been used as hosts for lanthanide ions, with doping occurring via site substitution.^44−51^ Such materials leverage the similar ionic radii and coordination chemistry of Bi^3+^ and the Ln^3+^ ions to achieve characteristic Ln^3+^ emission with just a fraction of the rare-earth elements required for an analogous homometallic Ln^3+^ phase. Furthermore, the heavy-atom effect imparted by bismuth has been exploited to realize efficient intersystem crossing and long-lived, room-temperature phosphorescence.^52^ For example, a series of phosphorescent bismuth-halide materials bridged through the rigid ligand, bipyrimidine, were reported with lifetimes as long as 11.36 ms at 77 K.^53^ The compounds were synthesized from ionic liquids and the resulting photophysical properties were tunable based on the ionic liquid employed as it incorporated into the structure. Related bismuth-halide materials have similarly shown phosphorescence originating from 2,2′-bipyridyl derivatives; these compounds exhibited long lifetimes and X-ray luminescence, with the latter alluding to the potential application of bismuth–organic materials as X-ray scintillators.^54,55^

Given the promising developments in bismuth-based luminescent materials design, our group has sought to further elucidate structure–property relationships in these phases. Of particular interest is the role that noncovalent interactions have in the photophysical properties of bismuth–organic materials. To understand these effects, our initial work was motivated by previous work in 2,6-pyridinedicarboxylate (PDC) ligand systems^56^ and specifically the observation of a common dimeric motif.^57−59^ We hypothesized that such units could be utilized to study the effects of noncovalent interactions between the bismuth–organic complexes and outer coordination sphere fluorophores on photophysical properties. Recently, we detailed the synthesis and characterization of a bismuth–organic structure in which stabilization of 1,10-phenanthrolinium (Hphen) via hydrogen bonding and π–π interactions led to long-lived room-temperature phosphorescence characteristic of Hphen triplet emission.^60^ Furthermore, in a subsequent report, we highlighted the impact of π–π interactions on luminescent properties in a series of analogous structures with phen-derivatives; phosphorescence from the phen-derivatives was only achieved when strong π–π interactions occurred between PDC and the phen-derivatives and in the absence of π–π interactions due to steric effects, weak fluorescence was observed.^61^ Thus, by exploiting strong π–π interactions, this system yields predictable phosphorescence with the emission dictated solely by the identity of the N-heterocycle employed during synthesis. Yet, the effect that hydrogen bonding had on the emissive properties remained unclear. To this end, in this work, we sought to understand the impact of hydrogen bonding on the phosphorescence of the outer sphere fluorophore. Herein, we describe the synthesis, structure, photoluminescent, and radioluminescent properties of three analogous compounds with the general formula [Bi_2_(HPDC)_2_(PDC)_2_]·[fluorophore]·2H_2_O, where H_2_PDC is 2,6-pyridinedicarboxylic acid and [fluorophore] is pyrene, naphthalene, and azulene. Notably, these fluorophores are PAHs incapable of hydrogen bonding and thus, together with previous work, provide important insights into the role of noncovalent interactions including hydrogen bonding and π–π stacking interactions on Bi–organic phosphorescence.

## Results

### Structure Descriptions

#### [Bi_2_(HPDC)_2_(PDC)_2_]·(Pyrene)·2H_2_O (**1**)

**Figure 1:**
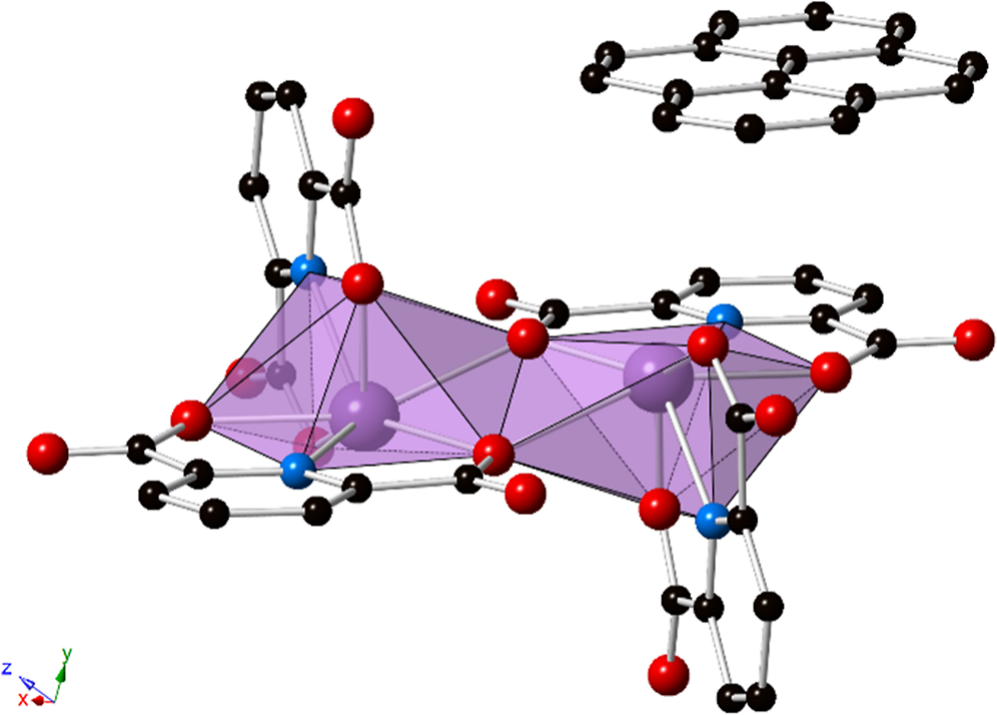
Polyhedral representation of **1**. The structure consists of Bi_2_(HPDC)_2_(PDC)_2_ dimers, with pyrene in the outer coordination sphere. Strong π–π stacking interactions exist between the bridging PDC and the pyrene. Purple = bismuth, blue = nitrogen, red = oxygen, and black = carbon atoms. Hydrogen atoms and lattice water molecules have been omitted for clarity.

The asymmetric unit of compound **1** consists of one crystallographically unique bismuth ion that is coordinated to a tridentate, doubly deprotonated PDC, a tridentate, singly deprotonated HPDC, half of a pyrene molecule, and one water molecule; application of the inversion symmetry about the pyrene yields one pyrene molecule per unit cell. As shown in Figure 1, the bismuth center is bridged by the doubly deprotonated PDC to a symmetry-equivalent site to form dimeric structural units. The bismuth metal centers are seven-coordinate and exhibit a hemidirected coordination geometry, with an open face indicative of a stereochemically active 6s^2^ lone pair. A pyrene molecule exists in the outer coordination sphere and occupies the space afforded by the open Bi coordination site. The Bi–O distances range from 2.189(3) to 2.655(3) Å and the Bi–N distances are 2.409(3) and 2.440(3) Å. The significant variance between Bi–O bond lengths is characteristic of the 6s^2^ stereochemically active lone pair, with the shortest bond occurring trans to the lone pair.

**Figure 2:**
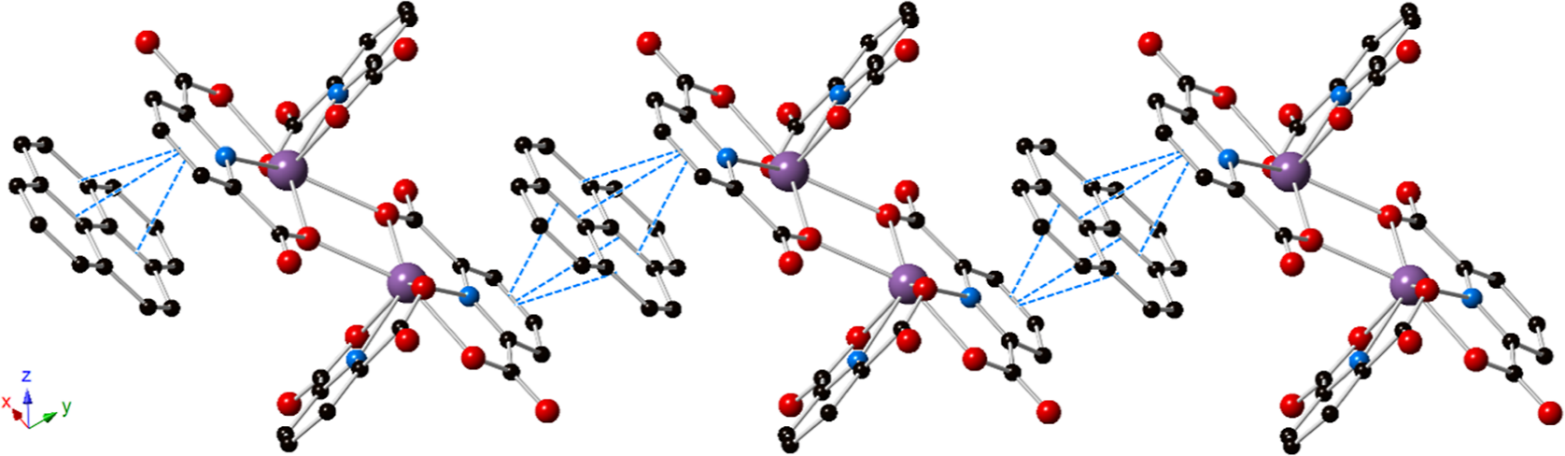
Ball and stick representation of **1** showing the 1-D chains formed by PDC···pyrene···PDC π–π stacking interactions (blue dotted lines, depicting centroid···centroid interactions). Purple = bismuth, blue = nitrogen, red = oxygen, and black = carbon atoms. Hydrogen atoms and lattice water molecules have been omitted for clarity.

As shown in Figure 2, π–π stacking interactions between the PDC of one dimer, the outer coordination pyrene, and a PDC of a dimer in the next unit cell (PDC···pyrene···PDC interactions) result in propagation of the dimeric units into 1-D supramolecular chains. The centroid···centroid distances are 3.569(3), 3.600(3), and 3.861(3) Å, with slip angles of 19.3, 20.8, and 29.2°, respectively. Additionally, there are O_HPDC_–H···O_water_ hydrogen-bonding interactions with a D_HPDC_···A_water_ distance of 2.493(5) Å and D–H···A angle of 178(8)°. Similarly, there are O_water_–H···O_PDC_ interactions with D_water_···A_PDC_ distances of 2.743(5) and 2.722(4) Å and angles of 162(5) and 168(4)°, respectively. The hydrogen-bonding interactions bridge the 1-D chains into an overall 3-D extended network (Figures S4 and S5). Furthermore, the closest Bi···centroid distance is 3.889 Å with a β angle of 22.2°, which is consistent with a lone-pair–π interaction between bismuth and the pyrene. Such lone-pair–π interactions have been noted to occur in some main group metals with an *n*s^2^ electron configuration (Sb^3+^, Bi^3+^, etc.).^62^ Additional nonclassical C–H···O interactions are also present between PDCs with a D–H···A distance of 3.216(5) Å and a bond angle of 127°.

#### [Bi_2_(HPDC)_2_(PDC)_2_]·(Naphthalene)·2H_2_O (**2**)

**Figure 3:**
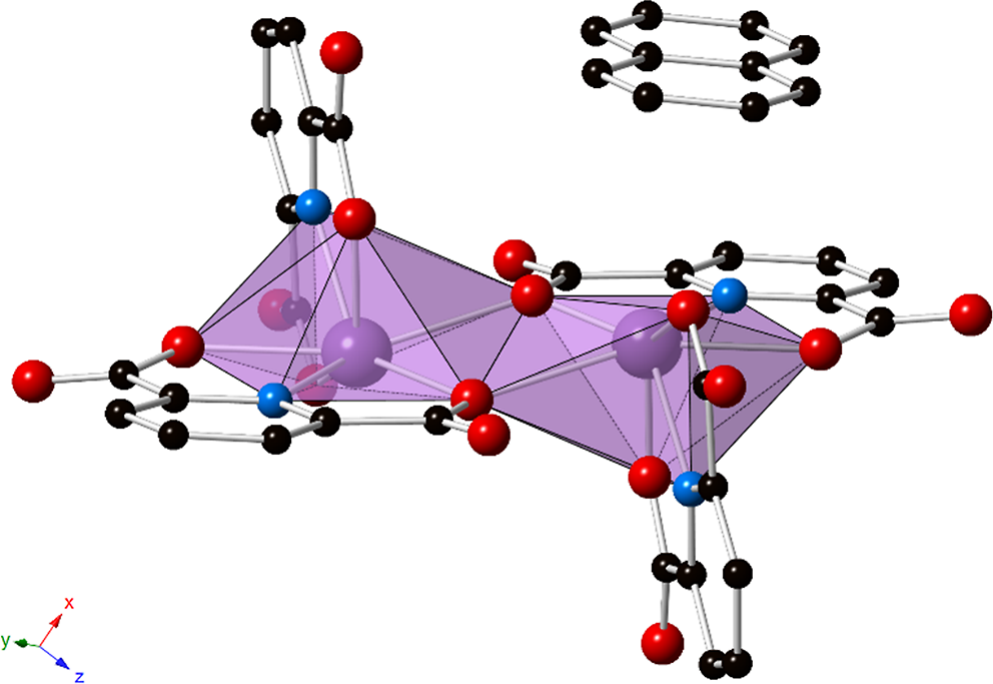
Polyhedral representation of **2**. The structure is built from the same Bi-PDC dimer as that observed in **1**; however, naphthalene is present in the outer coordination sphere and exhibits strong π–π stacking interactions with the bridging PDC. Purple = bismuth, blue = nitrogen, red = oxygen, and black = carbon atoms. Hydrogen atoms and lattice water molecules have been omitted for clarity.

The structure of **2** (Figure 3) is built from the same bismuth-PDC scaffold as that described for **1**.

The asymmetric unit consists of one crystallographically unique bismuth ion that is coordinated to a tridentate, doubly deprotonated PDC, a tridentate, singly deprotonated HPDC, half of a naphthalene molecule, and one water molecule; application of the inversion symmetry about the naphthalene yields one naphthalene molecule per unit cell. The bismuth metal center displays a hemidirected coordination environment with the stereochemically active 6s^2^ lone pair directed toward the large open coordination site. The Bi–O distances range from 2.180(4) to 2.705(4) Å and the Bi–N distances are 2.434(4) and 2.462(4) Å.

**Figure 4:**
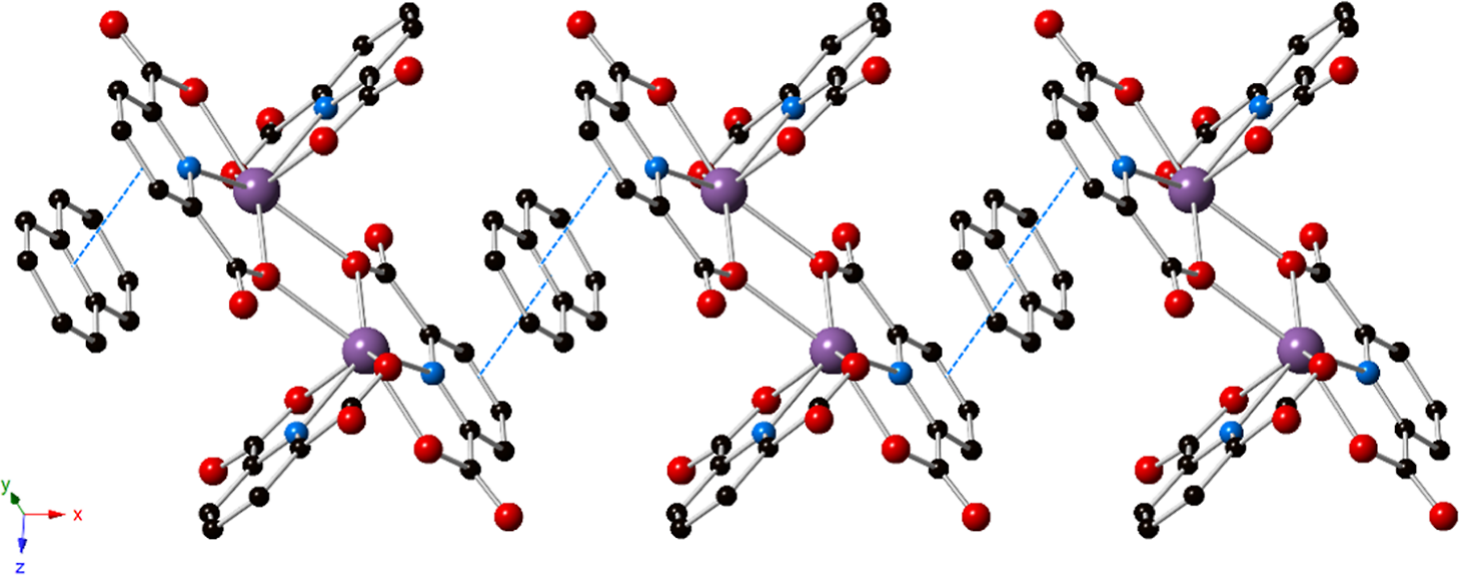
Ball and stick representation of **2** showing the 1-D chains that extend down the [100] by PDC···naphthalene···PDC π–π stacking interactions (blue dotted lines). Purple = bismuth, blue = nitrogen, red = oxygen, and black = carbon atoms. Hydrogen atoms and lattice water molecules have been omitted for clarity.

As shown in Figure 4, the structure of **2** consists of 1-D chains that extend down the [100] via π–π stacking interactions between PDC···naphthalene···PDC rings. The centroid···centroid distances are 3.556(4) and 3.555(4) Å with slip angles of 23.9°. These are effectively the same PDC···naphthalene interaction, generated through symmetry, and hence the two nearly identical interaction parameters. Additionally, there are O_HPDC_–H···O_water_ hydrogen-bonding interactions as in **1** with a D_HPDC_···A_water_ distance of 2.488(5) Å and D–H···A angle of 168(3)°, as well as O_water_–H···O_PDC_ interactions with D_water_···A_PDC_ distances of 2.755(5) and 2.741(5) Å and angles of 168(6) and 164(5)°, respectively. The hydrogen-bonding interactions bridge the 1-D chains into an extended 3-D network (Figures S6 and S7). Additionally, there exist potential LP–π interactions between bismuth and the naphthalene, with a Bi···centroid distance of 3.579 Å and β angle of 10.2°. Nonclassical C–H···O interactions are also present with distances of 3.185(6), 3.173(6), 3.366(6), and 3.458(7) Å and bond angles of 123, 127, 158, and 166°, respectively, although the latter two interactions are relatively long and weak.

#### [Bi_2_(HPDC)_2_(PDC)_2_]·(Azulene)·2H_2_O (**3**)

**Figure 5:**
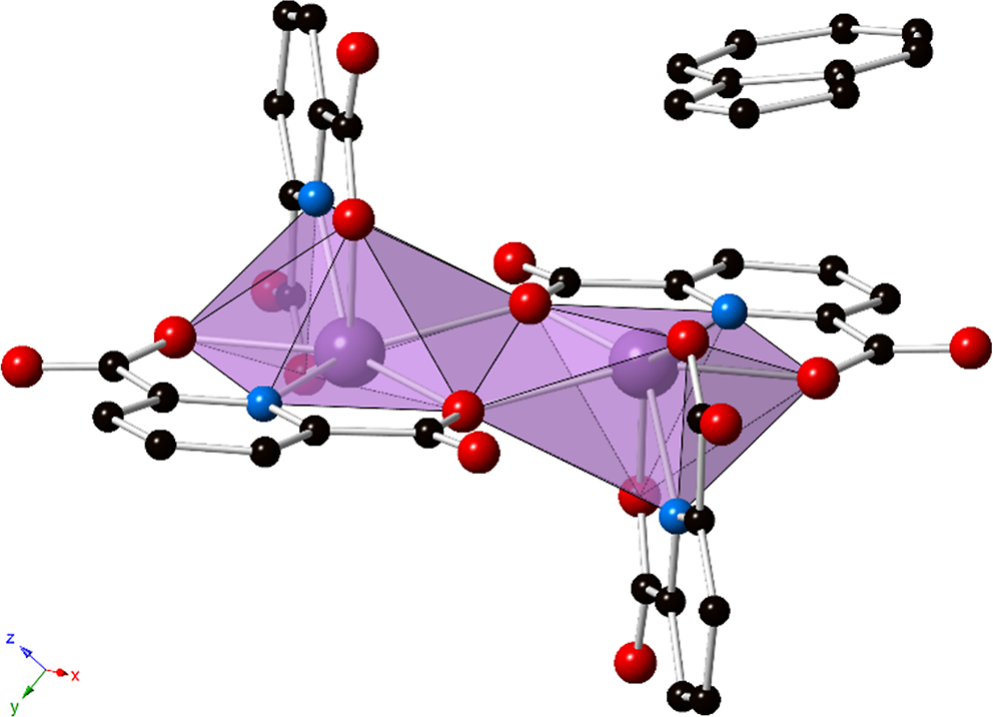
Polyhedral representation of **3**. Outer sphere azulene displays strong π–π stacking interactions with the bridging PDC. Purple = bismuth, blue = nitrogen, red = oxygen, and black = carbon atoms. Hydrogen atoms, lattice water molecules, and disorder of the azulene have been omitted for clarity.

Compound **3** is isomorphous with **2** and consists of the same [Bi_2_(HPDC)_2_(PDC)_2_] dimeric unit (Figure 5). The asymmetric unit consists of one crystallographically unique bismuth ion, a doubly deprotonated PDC, a singly deprotonated HPDC, half of an azulene molecule, and one water molecule; application of the inversion symmetry about the azulene yields one azulene molecule per unit cell. The bismuth ions are seven-coordinate with a hemidirected coordination geometry. The Bi–O distances range from 2.196(3) to 2.705(3) Å and the Bi–N distances are 2.438(3) and 2.454(4) Å.

**Figure 6:**
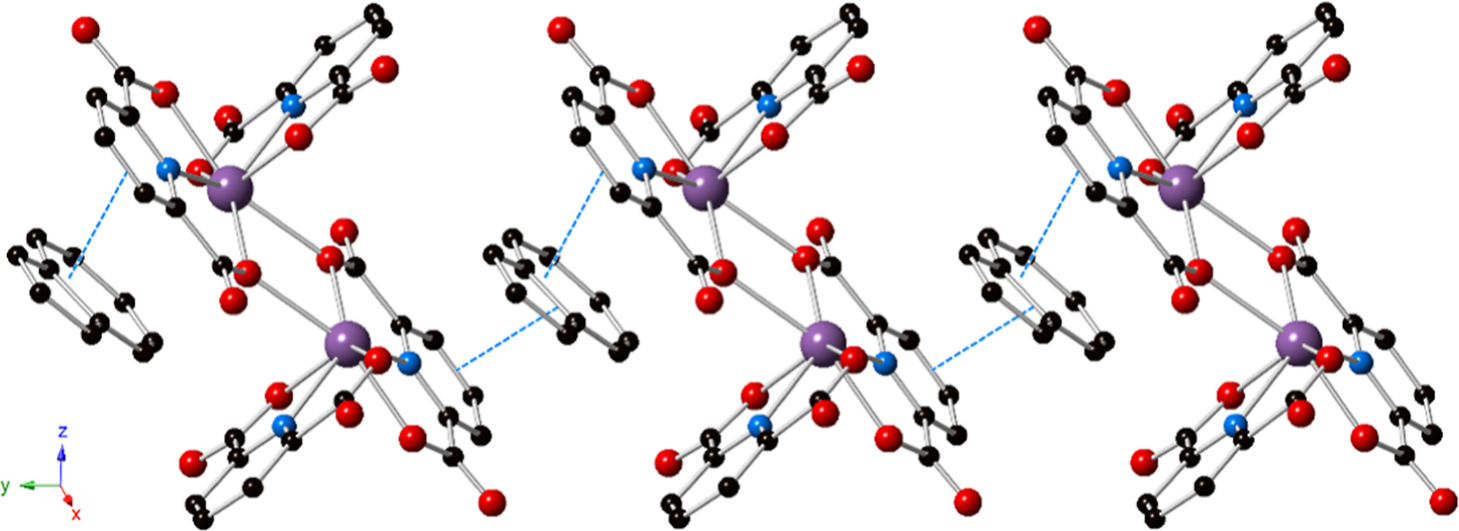
Ball and stick representation of **3** highlighting the 1-D chains formed via PDC···azulene···PDC π–π interactions (blue dotted lines). Purple = bismuth, blue = nitrogen, red = oxygen, and black = carbon atoms. Hydrogen atoms, lattice water molecules, and disorder of the azulene are not shown for clarity.

An azulene molecule is present in the outer coordination sphere. The azulene is disordered over two sites, accounting for the two orientations of the asymmetric aromatic hydrocarbon. As depicted in Figure 6, strong π–π stacking interactions between PDC···azulene···PDC rings result in supramolecular 1-D chains. The centroid···centroid distance, between PDC and the five-membered ring on azulene, is 3.369(4) Å with a slip angle of 14.8°. The 1-D chains are further connected into a 3-D supramolecular structure through hydrogen bonding, as shown in Figures S8 and S9. The O_HPDC_–H···O_water_ hydrogen-bonding interaction has a D_HPDC_···A_water_ distance of 2.482(5) Å and D–H···A angle of 165(6)°, and the O_water_–H···O_PDC_ interaction exhibits D_water_···A_PDC_ distances of 2.754(4) and 2.766(5) Å with angles of 167(6) and 156(6)°, respectively. Nonclassical C–H···O interactions may be present with a distance of 3.189(5) Å and bond angle of 126°.

### Photoluminescence

**Figure 7:**
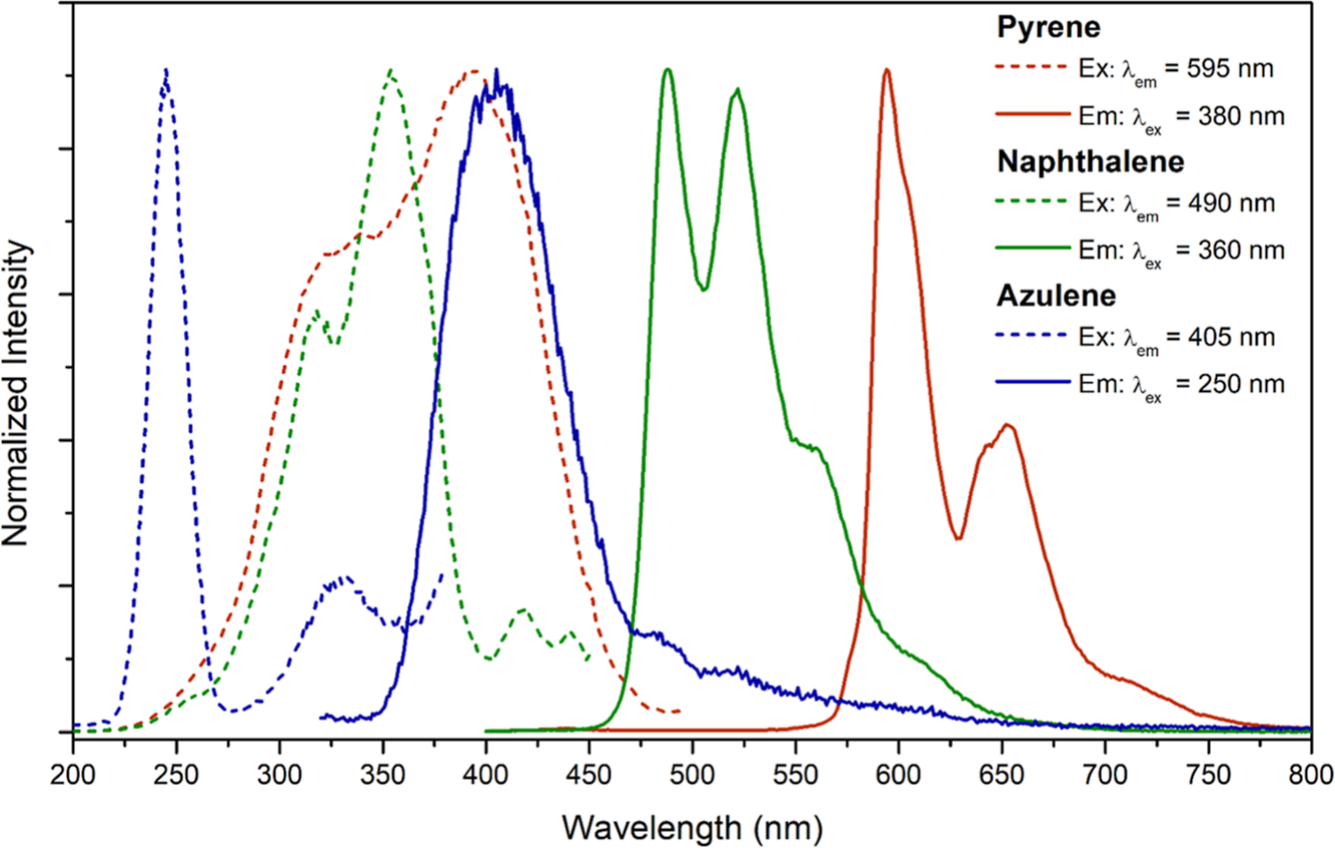
Normalized excitation (dashed lines) and emission (solid lines) spectra for pyrene (**1**; red), naphthalene (**2**; green), and azulene (**3**; blue).

All three compounds display room-temperature photoluminescence in the solid state of varying intensity; normalized excitation and emission plots for **1**–**3** are shown in Figure 7. The CIE 1931 coordinates were calculated for each emission spectrum and the chromaticity of each compound is presented in Figure S16. Compound **1** emits in the red region between 570 and 750 nm with a maximum at 595 nm (16,807 cm^–1^) upon excitation at 380 nm. The presence of additional local maxima at 655 nm (15,267 cm^–1^) and a smaller shoulder at 715 nm (13,986 cm^–1^) is attributed to vibronic coupling from an organic emitter, with peak separations of 1540 and 1281 cm^–1^, respectively. The excitation spectrum for the emission at 595 nm is very broad; there are two main peaks at 390 and 335 nm, and the profile extends into the visible region. The emission spectrum for **1** is consistent with pyrene phosphorescence and has been described previously, most commonly for solution-state studies that have examined host–guest properties.^63−69^ The luminescence decay spectrum for emission at 595 nm fit well with a biexponential decay function, indicating the presence of two distinct lifetimes of 465.5 and 128.9 μs (Figure S13). The shorter lifetime is attributed to direct singlet excitation of the pyrene molecule, followed by intersystem crossing and emission from the pyrene triplet state. The excitation spectrum matches well with that of pyrene in the solid state (Figure S17), providing evidence for partial contribution from direct excitation of pyrene. The solid-state quantum yield (Φ) for **1** was 1.3(2)%.

Compound **2** emitted intense green light between 450 and 750 nm upon excitation at 360 nm. The maximum emission wavelength was 490 nm (20,408 cm^–1^), and splitting resulted in additional local maxima at 520 (19,231 cm^–1^) and 555 nm (18,018 cm^–1^), again indicative of vibronic coupling from an organic emitter with coupling energies of 1177 and 1213 cm^–1^, respectively. The excitation spectrum was broad with two main peaks: a maximum around 360 nm and another peak centered at 320 nm. The luminescence decay spectrum for **2** recorded at 490 nm fit well with a single exponential decay function, yielding a lifetime of 485.5 μs (Figure S14). The emission profile is attributed to phosphorescence from the outer coordination naphthalene and is consistent with previous reports.^69−71^ The quantum yield for **2** was 30.8(4)%.

Compound **3** displayed very weak emission at 405 nm upon excitation at 250 nm; albeit the lamp was only weakly emitting at 250 nm and may contribute to the low emission intensity. Nonetheless, as compared to **1** and **2**, the emission spectrum does not exhibit splitting indicative of vibronic coupling. Rather, the emission spectrum exhibits a comparatively sharp band, with an excitation maximum around 250 nm and a significantly less intense peak centered at approximately 330 nm. The decay spectrum for the emission of **3** at 405 nm fit well with a single exponential decay function with a lifetime of 111.2 μs (Figure S15). Quantum yields were not collected for **3** given the prohibitively weak emission intensity.

### Radioluminescence

**Figure 8:**
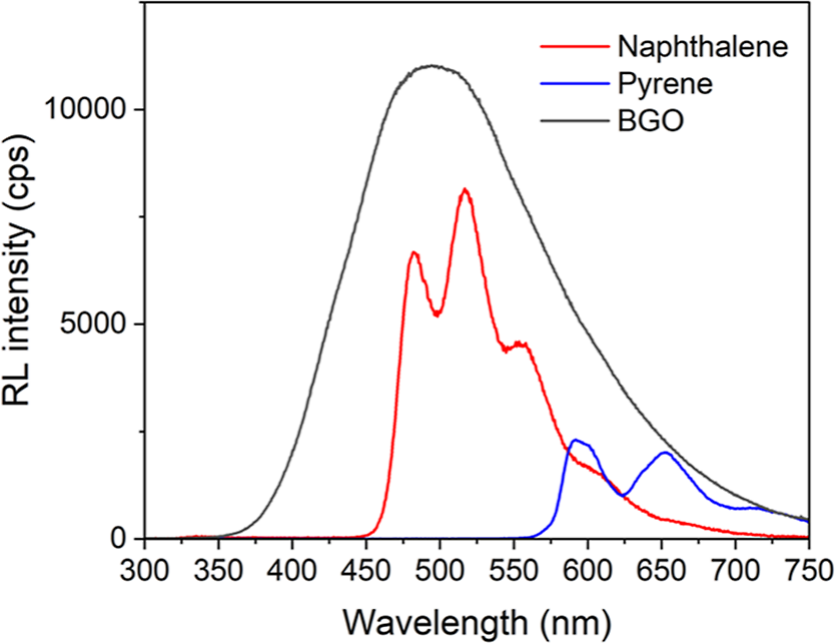
Radioluminescence spectra for **1** (blue), **2** (red), and BGO (black). The spectra were integrated and the relative intensities were compared to that of BGO.

Compounds **1** and **2** display luminescence upon irradiation with X-rays, as shown in Figure 8. Bismuth and other heavy atoms can absorb ionizing radiation and eject photoelectrons which may result in the emission of light in a process called X-ray luminescence or radioluminescence.^71−75^ The emission displayed from both compounds is effectively identical to that observed via photoexcitation; **1** shows characteristic pyrene phosphorescence and **2** shows characteristic naphthalene phosphorescence. This is consistent with a previous study wherein radioluminescence was observed from phosphorescent bismuth–organic compounds, with the emission dictated by the triplet-state energies of the outer coordination sphere phenanthroline derivatives employed.^61^

The integrated intensities of the radioluminescence spectra were compared to that of bismuth germanate (BGO), a common bismuth-based standard for radioluminescence intensity comparisons. Compound **1** displayed a relative intensity of 11% compared to BGO, while compound **2** displayed a relative intensity of 38% compared to BGO powder. While these relative intensities clearly pale in intensity to BGO, there is novelty in radioluminescence with tunable properties dictated by the organic arene ligand. Indeed, examples of bismuth–organic scintillators are limited.

## Discussion

Compounds **1**–**3** all display photoluminescence at room temperature in the solid state. Both **1** and **2** display phosphorescence characteristic of the outer coordination sphere arene molecules.^63−68,70,76^ Importantly, these compounds display strong π–π stacking interactions yet contain no hydrogen-bonding interactions with the outer coordination arene molecule. In a previous study, we reported a similar bismuth–organic dimer with an outer coordination N-heterocycle, Hphen; the compound exhibited characteristic Hphen phosphorescence at room temperature in the solid state.^60^ Much like compounds **1**–**3**, the Hphen engaged in strong π–π stacking interactions with the PDC of the bismuth complex but also acted as a hydrogen bond donor to a nitrate anion on the bismuth complex. The role that the noncovalent interactions had on the luminescence was not entirely clear. Absence of hydrogen bonding between the Bi complex and the outer sphere fluorophore in **1**–**3** shows that such H-bonding interactions have little effect on the lifetimes and efficiencies of these phosphorescent materials; compound **2** in particular displays a higher quantum yield (Φ = 30.8%) than that observed for the Hphen analogue (Φ = 27.4%).

Previously, we proposed that the outer coordination fluorophore was plausibly sensitized via triplet–triplet energy transfer.^61^ We proposed that protonation of the fluorophores (i.e., Hphen and 2,9-dimethyl-1,10-phenanthrolinium) allowed for a stepwise electron and hole transfer due to the presence of a metastable, neutrally charged intermediate state; when the fluorophore was not ionized (i.e., 2,9-dichloro-1,10-phenanthroline), the sensitization could occur through a concerted energy-transfer mechanism, leading to shorter lifetimes. The work presented herein is further consistent with a concerted energy-transfer mechanism. Both **1** and **2** display lifetimes (465.5 and 485.5 μs, respectively) consistent with the previously reported neutrally charged [Bi_2_(HPDC)_2_(PDC)_2_(H_2_O)]·(Cl_2_Phen) compound which showed Cl_2_Phen phosphorescence, with a lifetime of 534 μs. The presence of strong π–π stacking interactions appears to be the defining structural feature to achieve phosphorescence in this system, and the presence or absence of an ionized fluorophore controls the lifetime.

The efficiency of the emission is likely dictated by the energy of the emitting T_1_ state of the fluorophore relative to the bismuth complex. This is consistent with the inefficient emission (Φ = 1.3%) observed for **1**, which likely results from the significant Stoke’s shift of the material; excitation occurred at 380 nm and the emission maximum was centered at 595 nm. Compound **2**, by comparison, had an excitation maximum of 360 nm and an emission maximum of 490 nm. This suggests that inefficient energy transfer occurs between the bismuth complex and pyrene; there is poor energy matching between the donor and acceptor excited states resulting in significant nonradiative relaxation. A similar trend can be observed in the radioluminescence intensities; **1** displayed a relative intensity to BGO of 11%, while **2** displayed a relative intensity of 38%. This is likely the result of inefficient energy transfer to the pyrene molecule compared to naphthalene. These values are consistent with a previous report wherein bismuth complexes synthesized with 2,9-dimethyl-1,10-phenanthroline and 2,9-dichloro-1,10-phenanthroline displayed radioluminescence with relative intensities of 33 and 52% compared to BGO powder, respectively.^61^

Azulene was chosen as an outer coordination arene molecule due to its unique optical and electronic properties.^77^ Azulene was the first molecule discovered to display S_2_ → S_0_ emission,^78^ breaking Kasha’s rule which states that “*the emitting electronic level of a given multiplicity is the lowest excited level of the multiplicity*”.^79^ While T_2_ → S_0_ emission has been reported from organic molecules before, it is rare and to the best of our knowledge has not been reported for azulene.^80^ This system, which achieves triplet emission from the outer coordination arene ligand, seemed like the ideal opportunity to achieve azulene phosphorescence. While the long lifetime of **3** (111.2 μs) is consistent with phosphorescence, the emission profile of **3** does not exhibit features indicative of vibronic coupling as observed for **1** and **2**. The peak at 405 nm is slightly red-shifted from previous reports of S_2_ → S_0_ emission from azulene, with peaks around 375 and 390 nm, again consistent with a red shift expected for T_2_ → S_0_ emission. However, the shift is small.^81^ Thus, the origin of the emission at 405 nm remains unclear, with several feasible pathways including azulene, the bismuth complex, or some charge-transfer state on the whole bismuth–organic structure. It should be noted that the emission intensity of **3** is very weak—no significant emission can be observed with the naked eye.

## Conclusions

Three novel bismuth–organic complexes with arenes (pyrene, naphthalene, and azulene) in the outer coordination sphere were synthesized. These hydrocarbons were stabilized via strong π–π stacking interactions between the PDC of the bismuth complex and the arene. Compounds **1** and **2** containing pyrene and naphthalene, respectively, displayed characteristic phosphorescence and radioluminescence from the outer coordination sphere arene molecule while **3**, the azulene analogue, displayed very weak emission from an unknown high-energy excited state. This work follows previous reports wherein phosphorescence from N-heterocycles was achieved in a similar manner, but importantly, this work highlights that the presence of hydrogen bonding has little effect on the lifetimes or efficiencies of the phosphorescence. Similarly, this work promotes the possibility of achieving phosphorescence from a much larger catalog of arene ligands, allowing for the tunability of emissive properties simply by changing the fluorophore identity.

## Experimental Methods

### Materials

Bi(NO_3_)_3_·5H_2_O (Fisher, 99.2%), 2,6-pyridinedicarboxylic acid (Acros Organics, 99%), nitric acid (Sigma-Aldrich, 70%), azulene (Alfa Aesar, 99%), pyrene (Aldrich, 99%), naphthalene (Aldrich, ≥99%), 2-propanol (Fisher Chemical), methylene chloride (Fisher Chemical), and ethanol (Fisher Chemical, 90%) were used as received. Nanopure water was used for all experiments (≤0.05 μS; Millipore, USA).

### Synthesis

#### [Bi_2_(HPDC)_2_(PDC)_2_]·(Pyrene)·2H_2_O (**1**)

2,6-Pyridinedicarboxylic acid (0.0334 g; 0.20 mmol), pyrene (0.0051 g; 0.025 mmol), and ethanol (1.5 mL) were added to a 7.5 mL glass vial and sonicated for 5 min in a bath sonicator. Bismuth nitrate pentahydrate (0.0243 g; 0.05 mmol) was dissolved in 2 M nitric acid (0.5 mL) in a separate vial and diluted with an additional aliquot of water (1 mL). The ethanolic solution was then gently layered on top of the aqueous solution. The resulting mixture was capped and allowed to sit at room temperature. After 2 days, yellow plate-like crystals that exhibited red luminescence upon UV irradiation had precipitated on the bottom of the vial. The mother liquor was decanted and the crystals were rinsed with water, ethanol, and two aliquots of dichloromethane. A phase-pure bulk powder was isolated using the same method by stirring the ethanolic and aqueous solutions instead of layering, followed by gravity filtration of the product. Yield = 64% (based on Bi). Elemental analysis for C_44_H_28_Bi_2_N_4_O_18_ Calcd (Obs.): C, 40.08 (39.76); H, 2.14 (2.25); N, 4.25 (4.25%).

#### [Bi_2_(HPDC)_2_(PDC)_2_]·(Naphthalene)·2H_2_O (**2**)

2,6-Pyridinedicarboxylic acid (0.0334 g; 0.20 mmol), naphthalene (0.0768 g; 0.60 mmol), and 2-propanol (1.5 mL) were added to a 7.5 mL glass vial and sonicated for 10 min in a bath sonicator. In a separate vial, bismuth nitrate pentahydrate (0.0243 g; 0.05 mmol) was dissolved in 2 M nitric acid (0.5 mL) and diluted with an additional aliquot of water (1 mL). The ligand solution was then gently layered on top of the aqueous solution. The resulting mixture was capped and allowed to sit at room temperature. After 3 days, colorless plate-like crystals that exhibited green luminescence upon UV irradiation were observed. The mother liquor was decanted and the crystals were rinsed with water, ethanol, and two aliquots of dichloromethane. Yield = 75% (based on Bi). Elemental analysis for C_38_H_26_Bi_2_N_4_O_18_ Calcd (Obs.): C, 36.67 (36.57); H, 2.11 (2.21); N, 4.50 (4.41%).

#### [Bi_2_(HPDC)_2_(PDC)_2_]·(Azulene)·2H_2_O (**3**)

Compound **3** was synthesized using the same method as that described for **2**, replacing naphthalene with azulene (0.0256 g; 0.20 mmol). Dark-blue plate-like crystals were obtained as a pure phase. Yield = 83% (based on Bi). Elemental analysis for C_38_H_26_Bi_2_N_4_O_18_ Calcd (Obs.): C, 36.67 (37.02); H, 2.11 (2.25); N, 4.50 (4.23%).

### Single-Crystal X-ray Diffraction

Single crystals of **1**–**3** were isolated from the bulk samples, coated in N-paratone, and mounted on a MiTeGen micromount. Single-crystal X-ray diffraction data were collected on a Bruker D8 QUEST diffractometer at 100(2) K using an IuS X-ray source (Mo Kα radiation; λ = 0.71073 Å) and a Photon 100 detector. The diffraction data were integrated utilizing the SAINT software within APEX3.^82,83^ A multiscan absorption correction method was applied in SADABS.^84^ Intrinsic phasing was used to solve all three structures in SHELXT and structures were refined through full-matrix least-squares on F2 with the SHELXL software in shelXle64.^85,86^ Details of the refinement are provided in Table 1.

**Table 1:** Crystallographic Refinement Details for **1–3**

	1	2	3
MW (g/mol)	1318.66	1244.59	1244.59
*T* (K)	100(2)	100(2)	100(2)
λ (K α)	0.71073	0.71073	0.71073
μ (mm^–^^1^)	8.775	9.686	9.660
crystal system	triclinic	triclinic	triclinic
space group	*P*1̅	*P*1̅	*P*1̅
*a* (Å)	9.2325(2)	9.3216(3)	9.2435(7)
*b* (Å)	11.1406(3)	9.3590(3)	9.6020(7)
*c* (Å)	11.2691(3)	11.9812(3)	11.7706(9)
α (deg)	76.880(1)	111.959(2)	92.797(2)
β (deg)	71.456(1)	93.182(2)	110.752(2)
γ (deg)	68.088(1)	106.182(2)	107.347(2)
volume (Å^3^)	1011.76(5)	915.92(5)	918.43(12)
*Z*	1	1	1
*R*_int_	0.0348	0.0442	0.0529
*R* (*I* > 2σ)	0.0216	0.0304	0.0215
*wR*_2_	0.0461	0.0636	0.0538
GooF	1.107	1.030	1.064
residual density max and min (e/Å^3^)	0.96 and −0.74	1.56 and −1.17	2.10 and −1.49
CCDC no.	2252259	2252257	2252258

### Photoluminescence

Luminescence spectra were collected on a Horiba PTI QM-400 fluorometer on ground samples of **1**–**3** pressed between two quartz microscope slides. Wavelengths of 380, 360, and 250 nm were used to excite the samples, representing the maximum excitation wavelengths of **1**–**3**, respectively. Spectral widths of 3, 1, and 7 nm were used for **1**–**3**, respectively. Luminescence spectra for pyrene and naphthalene were collected using similar conditions (Figures S17 and S18). Lifetime measurements were collected using a Xenon pulse lamp source with a 100 Hz lamp frequency. Decay spectra were fit with exponential decay functions using the OriginPro 8.5 software. A 400 nm long-pass filter was utilized to prevent harmonic peaks resulting from the excitation source for **1** and **2**, and a 320 nm long-pass filter was utilized for **3**. CIE chromaticity coordinates were calculated from the emission spectra using the PTI FelixGX software. Quantum yield measurements were collected in triplicate with 2 nm spectral widths for **1** and **2**. Crystals of **1** and **2** were ground in dry KBr (1:50 ratio of sample/KBr) and placed in a Teflon powder holder under ambient conditions. The holder was then placed within an 8.9 cm integrating sphere with a Spectralon fluoropolymer coating. Blank absorption and emission reference spectra were collected using the Teflon sample holder filled with dried KBr.

### Radioluminescence

Radioluminescence measurements were carried out using a customer-designed configuration of the Freiberg Instruments Lexsyg Research spectrofluorometer equipped with a Varian Medical Systems VF-50J X-ray tube with a tungsten target. The X-ray source was coupled with a Crystal Photonics CXD-S10 photodiode for continuous radiation intensity monitoring. The light emitted by the sample was collected by an Andor Technology SR-OPT-8024 optical fiber connected to an Andor Technology Shamrock 163 spectrograph coupled to a cooled (−80 °C) Andor Technology DU920P-BU Newton CCD camera (spectral resolution of ∼0.5 nm/pixel). Radioluminescence was measured under continuous X-ray irradiation (W lines and bremsstrahlung radiation; 40 kV, 1 mA) with an integration time of 1 or 5 s. Powders filled ∼8 mm diameter, 0.5 mm deep cups thus allowing for relative radioluminescence intensity comparison between different samples. BGO powder [Alfa Aesar Puratronic, 99.9995% (metal basis)] was used as a reference. Spectra were automatically corrected using the spectral response of the system determined by the manufacturer. Comparison was done by integrating the spectra from 350 to 750 nm, with percentages being given in relation to the integrated signal of the BGO powder.

### Characterization Methods

Powder X-ray diffraction data were collected on ground samples of compounds **1**–**3** (Cu Kα; λ = 1.542 Å) on a Rigaku Miniflex diffractometer from 3 to 40° 2θ with a step speed of 1°/min (Figures S10–S12). Combustion elemental analysis data were collected on the bulk phases of **1**–**3** with a PerkinElmer Model 2400 Elemental Analyzer. Thermogravimetric analysis was collected using a TA Instruments Q50 Thermogravimetric Analyzer with flowing N_2_ with a temperature ramp rate of 5 °C/min (Figures S19–S21).

## Supporting Information Available

Crystallographic refinement details, thermal ellipsoid plots, packing diagrams, powder X-ray diffraction patterns, phosphorescence decay plots, and a table of supramolecular interactions (PDF)
