# Supplementary material for: Unlocking Arene Phosphorescence in Bismuth–Organic Materials
Source: Inorg Chem. 2024 Jun 1;63(24):11053–62. doi: 10.1021/acs.inorgchem.4c00606 (PMC11186004; doi:10.1021/acs.inorgchem.4c00606)
Supplement: Supplementary file 1 — ic4c00606_si_001.pdf [file ic4c00606_si_001.pdf]

Unlocking arene phosphorescence in bismuth-organic materials

*Alexander C. Marwitz,[1] Anuj K. Dutta,[1] Robin L. Conner,[2] Lulio A. Sanz,[1] Luiz G. Jacobsohn,[2] Karah E. Knope[1,]**

1 Department of Chemistry, Georgetown University, Washington, D.C. 20057, United States of America
2 Department of Materials Science and Engineering, Clemson University, Clemson, SC 29634, United States of America

* Corresponding author: Karah E. Knope, kek44@georgetown.edu

Supporting Information

I. Crystallographic Refinement Details 2
II. Thermal Ellipsoid Plots 2
III. Packing Diagrams Highlighting Supramolecular Interactions 5
IV. Powder X-Ray Diffraction Patterns 8
V. Lifetime Measurements 10
VI. Ligand Photoluminescence Spectra 12
VII. Thermogravimetric Analysis 13
VIII. Summary of Supramolecular Interactions 14
IX. References 16

I. Crystallographic Refinement Details

All crystallographic details can be found in the refinement section of the corresponding crystallographic information file (CIF). For **1-3**, all non-hydrogen atoms were refined anisotropically after being located in the Fourier difference map. Hydrogens involved with hydrogen bonding (i.e. O-H bonds for carboxylic acids and lattice waters) were located in the difference map. All O-H distances in carboxylic acids were fixed at 0.86 Å, and O-H distances in water molecules were fixed at 0.88 Å. U_{eq} values for H atoms on protonated oxygens were assigned as 1.5 times their carrier atom; remaining H atom U's were assigned as 1.2 times carrier U_{eq}.

For compound **3**, the azulene molecule was disordered over two positions with a total sum of 50% occupancy to account for the asymmetry of the molecule. Additionally, a negative PART command was applied due to the disorder occurring on a symmetry site, thus preventing incorrect bond formation between azulene and itself upon application of the inversion center symmetry in the *P*-1 space group.

II. Thermal Ellipsoid Plots

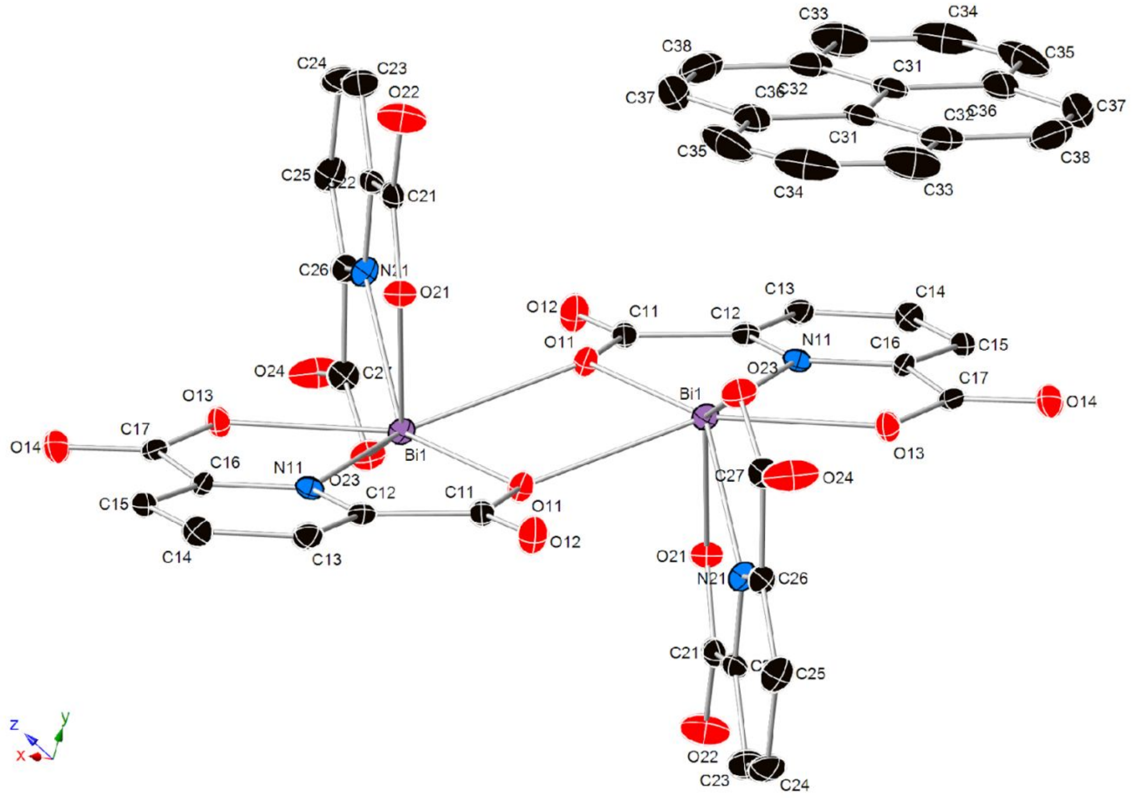

Figure S1. Thermal ellipsoid plot of **1** at 100 K. Ellipsoids are shown at 50% probability. Hydrogen atoms are not shown for clarity. Purple = bismuth; red = oxygen; blue = nitrogen; black = carbon atoms.

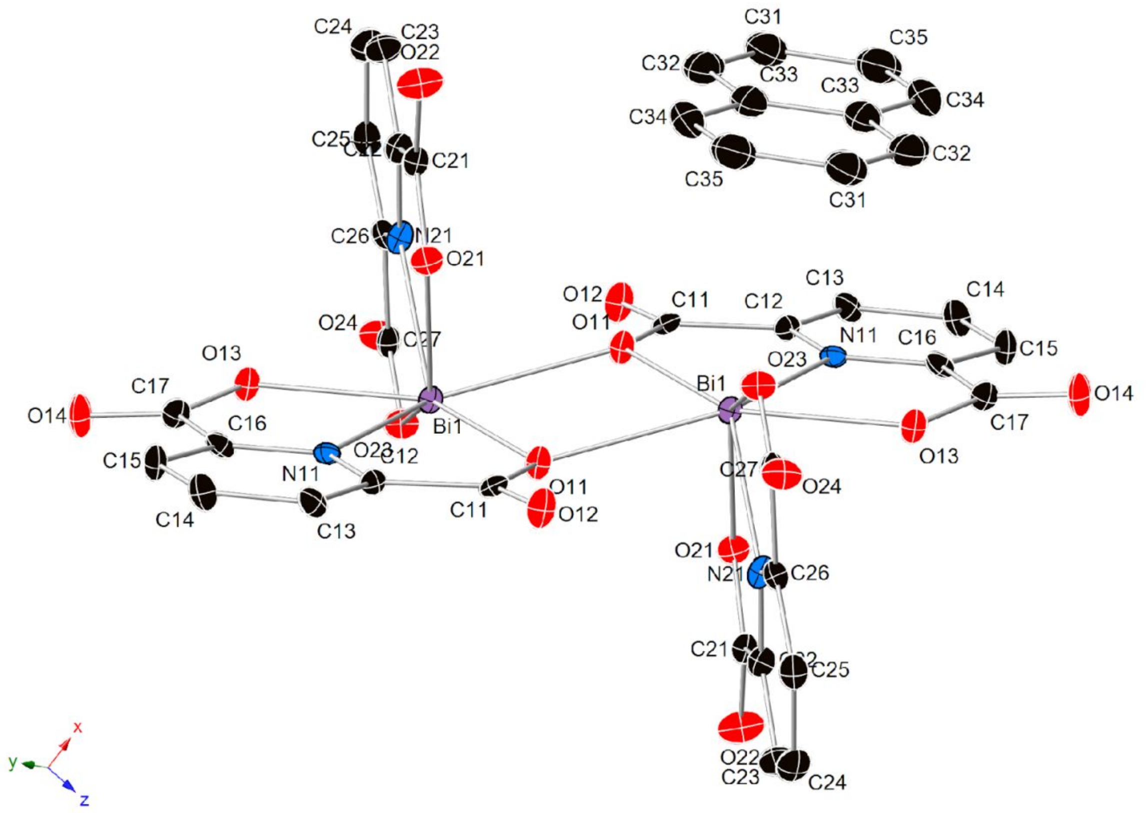

Figure S2. Thermal ellipsoid plot of **2** at 100 K. Ellipsoids are shown at 50% probability. Hydrogen atoms are not shown for clarity. Purple = bismuth; red = oxygen; blue = nitrogen; black = carbon atoms.

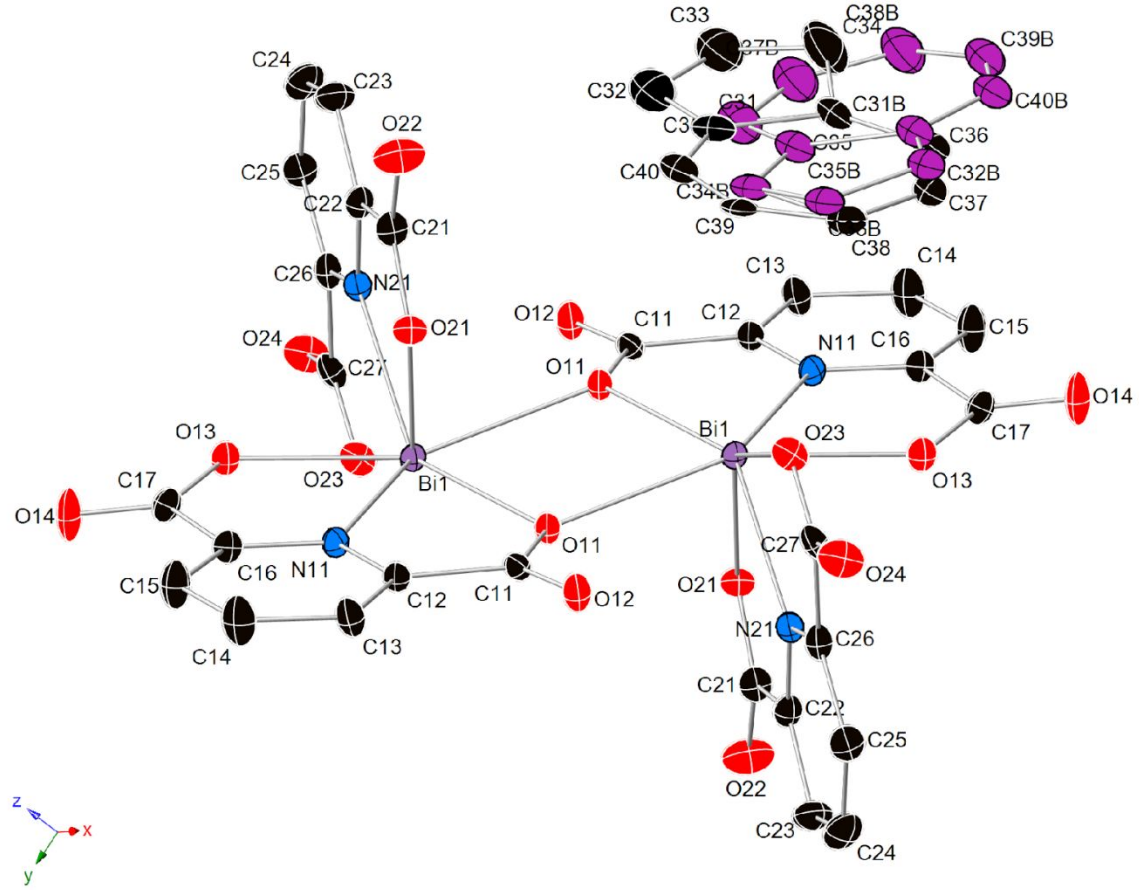

Figure S3. Thermal ellipsoid plot of **3** at 100 K. Ellipsoids are shown at 50% probability. Hydrogen atoms are not shown for clarity. Purple = bismuth; red = oxygen; blue = nitrogen; black = carbon atoms. The two positions of the disordered azulene is shown, with the carbons of one azulene position highlighted in magenta to improve clarity.

III. Packing Diagrams Highlighting Supramolecular Interactions

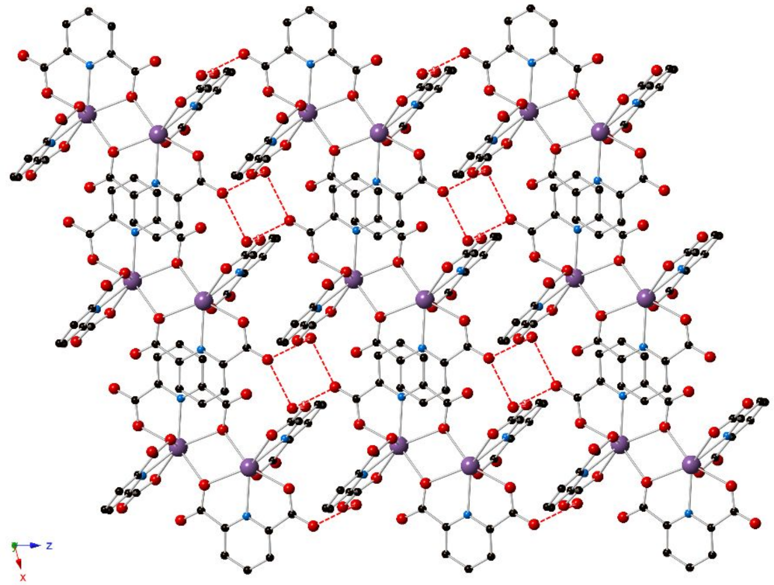

Figure S4. Packing diagram of **1** viewed down [010]. Hydrogen bonds are drawn as red dashed lines between donor atoms and acceptor atoms. 2-D Sheets are formed through hydrogen bonding interactions. Pyrene molecules are not shown to reduce clutter, as they are incapable of forming hydrogen bonds. Hydrogen atoms are not shown for clarity. Purple = bismuth; red = oxygen; blue = nitrogen; black = carbon atoms.

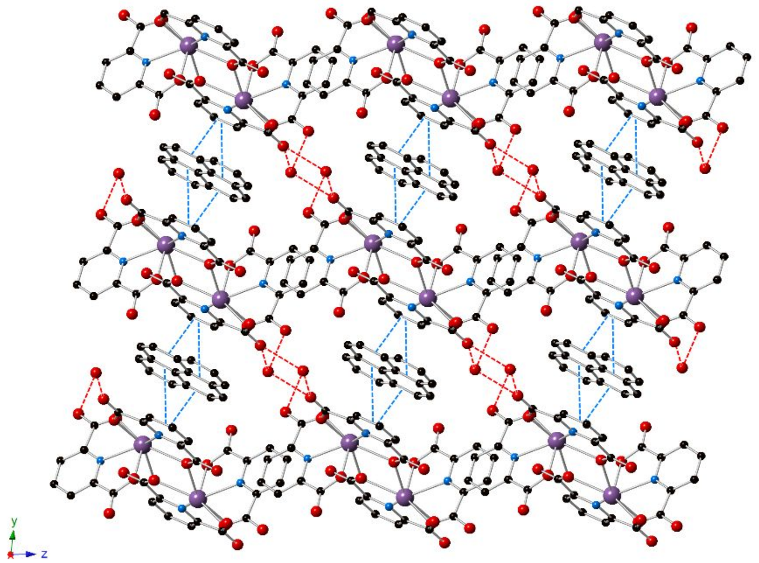

Figure S5. Packing diagram of **1** viewed down [100]. Hydrogen bonds are drawn as red dashed lines between donor atoms and acceptor atoms and π-π interactions are drawn as blue dashed lines between centroids. A 3-D supramolecular structure is formed through these interactions. Hydrogen atoms are not shown for clarity. Purple = bismuth; red = oxygen; blue = nitrogen; black = carbon atoms.

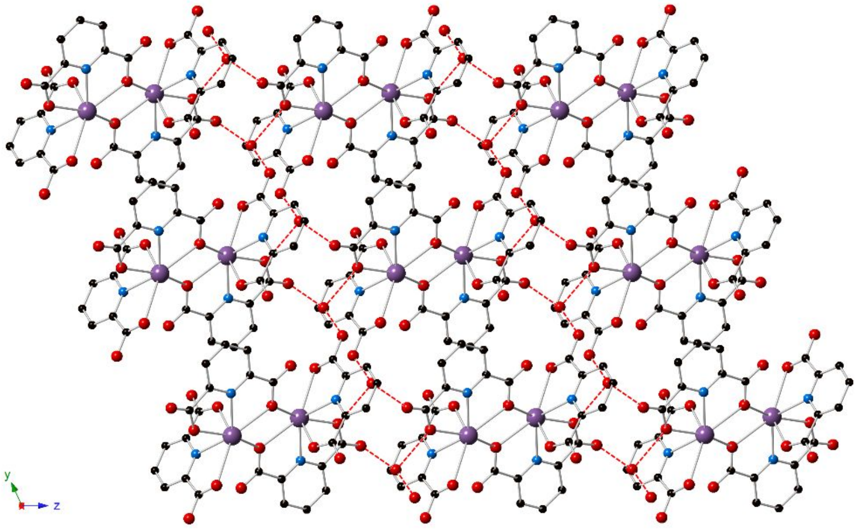

Figure S6. Packing diagram of **2** viewed down [100]. Hydrogen bonds are drawn as red dashed lines between donor atoms and acceptor atoms. 2-D Sheets are formed through hydrogen bonding interactions. Naphthalene molecules are not shown to reduce clutter, as they are incapable of forming hydrogen bonds. Hydrogen atoms are not shown for clarity. Purple = bismuth; red = oxygen; blue = nitrogen; black = carbon atoms.

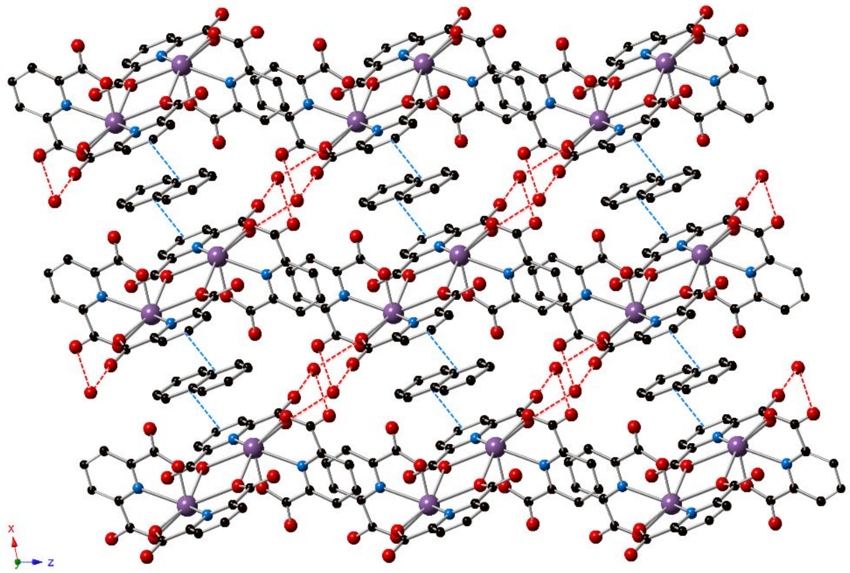

Figure S7. Packing diagram of **2** viewed down [010]. Hydrogen bonds are drawn as red dashed lines between donor atoms and acceptor atoms and π-π interactions are drawn as blue dashed lines between centroids. A 3-D supramolecular structure is formed through these interactions. Hydrogen atoms are not shown for clarity. Purple = bismuth; red = oxygen; blue = nitrogen; black = carbon atoms.

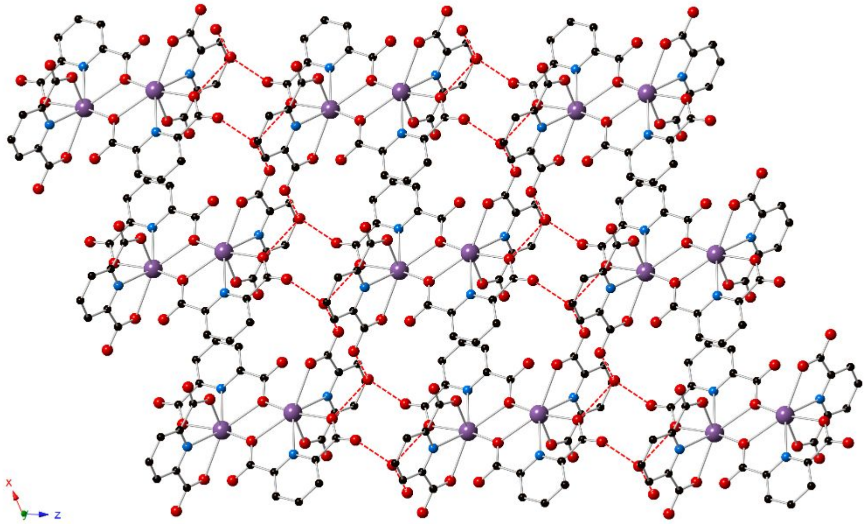

Figure S8. Packing diagram of **3** viewed down [010]. Hydrogen bonds are drawn as red dashed lines between donor atoms and acceptor atoms. 2-D Sheets are formed through hydrogen bonding interactions. Azulene molecules are not shown to reduce clutter, as they are incapable of forming hydrogen bonds. Hydrogen atoms are not shown for clarity. Purple = bismuth; red = oxygen; blue = nitrogen; black = carbon atoms.

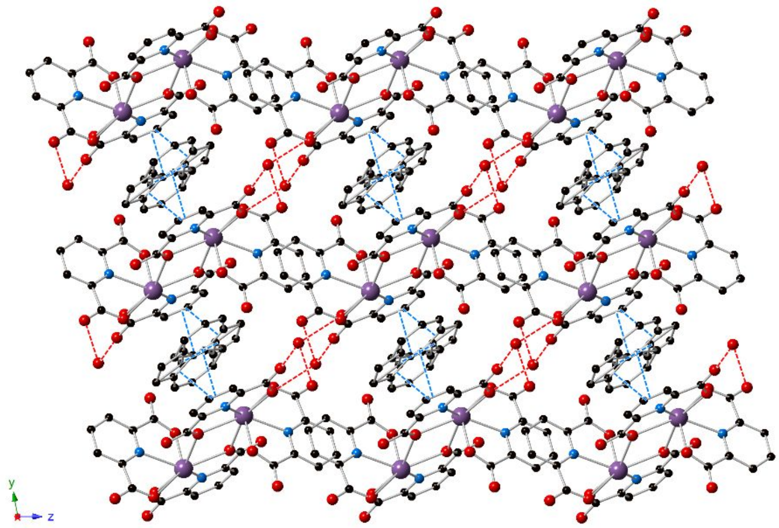

Figure S9. Packing diagram of **3** viewed down [100]. Hydrogen bonds are drawn as red dashed lines between donor atoms and acceptor atoms and π-π interactions are drawn as blue dashed lines between centroids. A 3-D supramolecular structure is formed through these interactions. Hydrogen atoms are not shown for clarity. Purple = bismuth; red = oxygen; blue = nitrogen; black = carbon atoms. The disorder of the azulene molecules can be seen as overlapping rings in opposite orientations.

IV. Powder X-Ray Diffraction Patterns

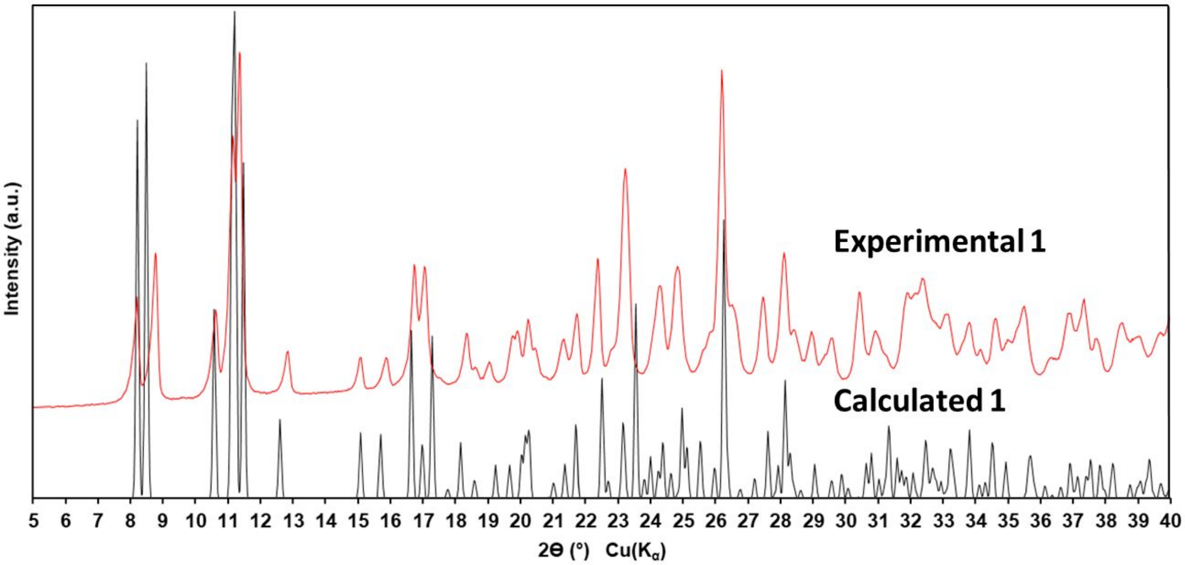

Figure S10. Experimental Powder X-ray Diffraction pattern for **1** (red) overlaid with the pattern calculated from the single crystal data collected at 100 K (black).

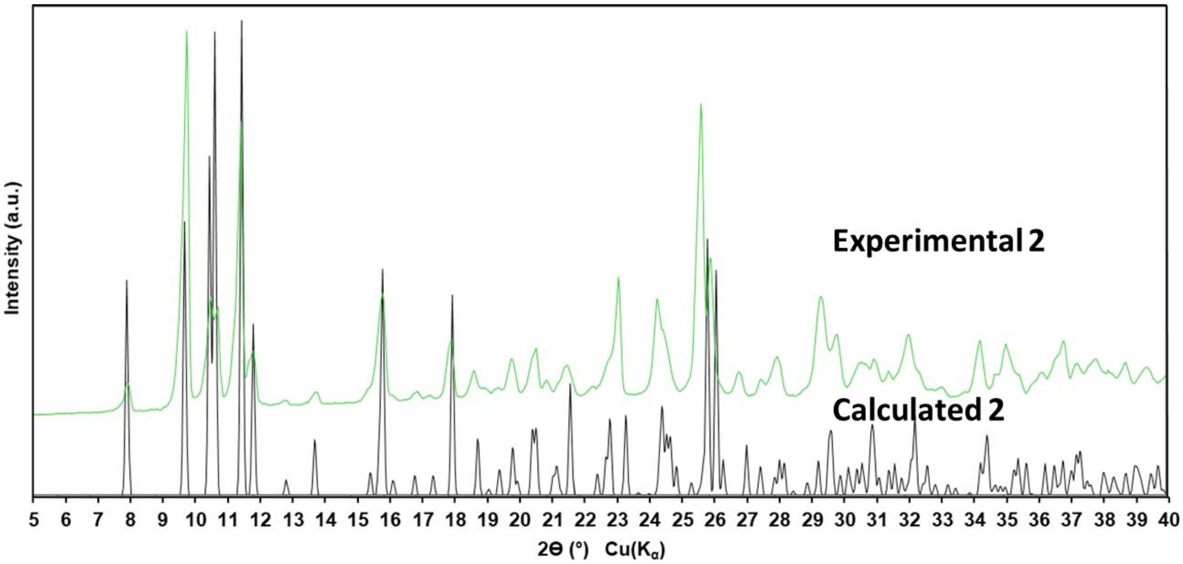

Figure S11. Experimental Powder X-ray Diffraction pattern for **2** (green) overlaid with the pattern calculated from the single crystal data collected at 100 K (black).

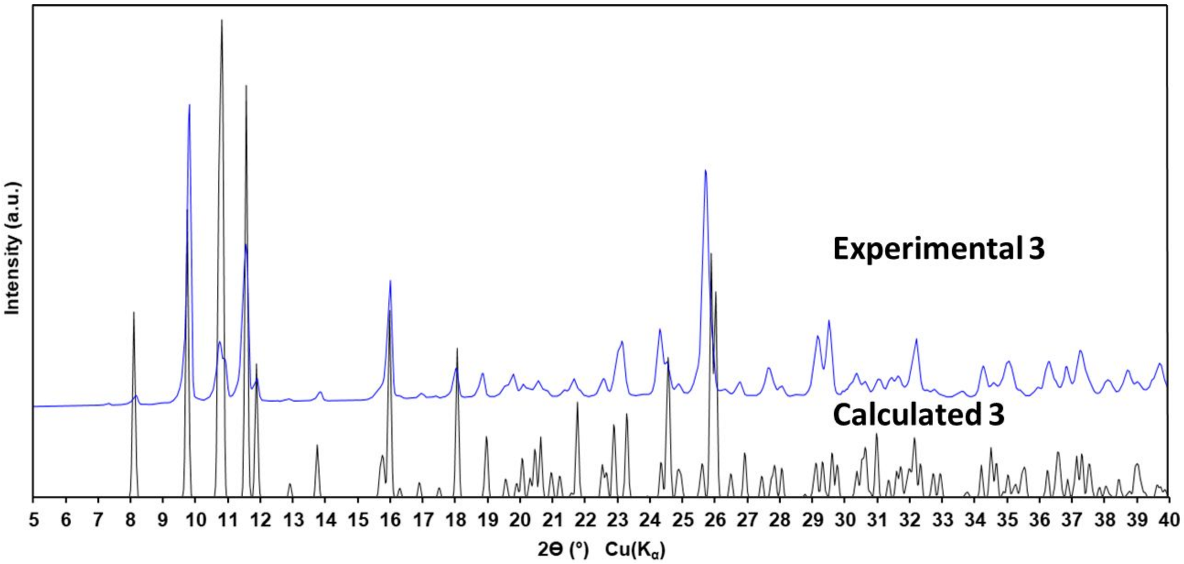

Figure S12. Experimental Powder X-ray Diffraction pattern for **3** (blue) overlaid with the pattern calculated from the single crystal data collected at 100 K (black).

V. Lifetime Measurements

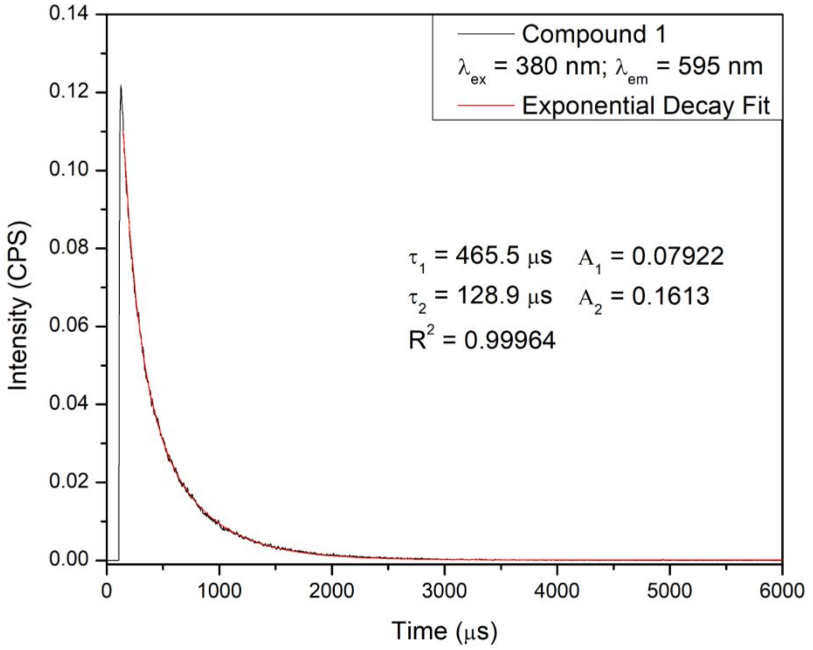

Figure S13. Experimental phosphorescence decay plot of **1** (black) fit with a biexponential decay function (red). A_1 and A_2 are the pre-exponential factors for τ_1 and τ_2, respectively. The excitation wavelength was 380 nm and the emission wavelength was 595 nm.

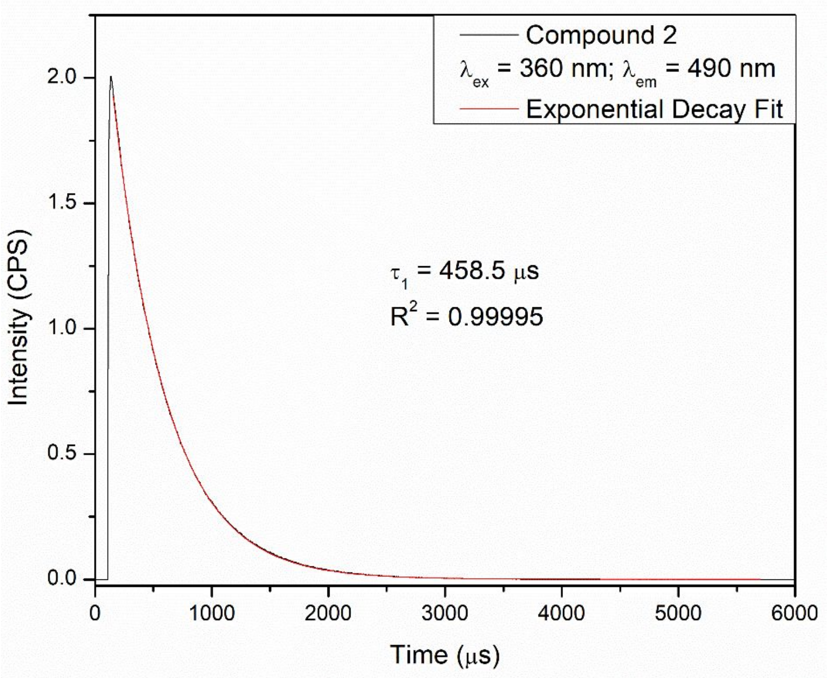

Figure S14. Experimental phosphorescence decay plot of **2** (black) fit with a single exponential decay function (red). The excitation wavelength was 360 nm and the emission wavelength was 490 nm.

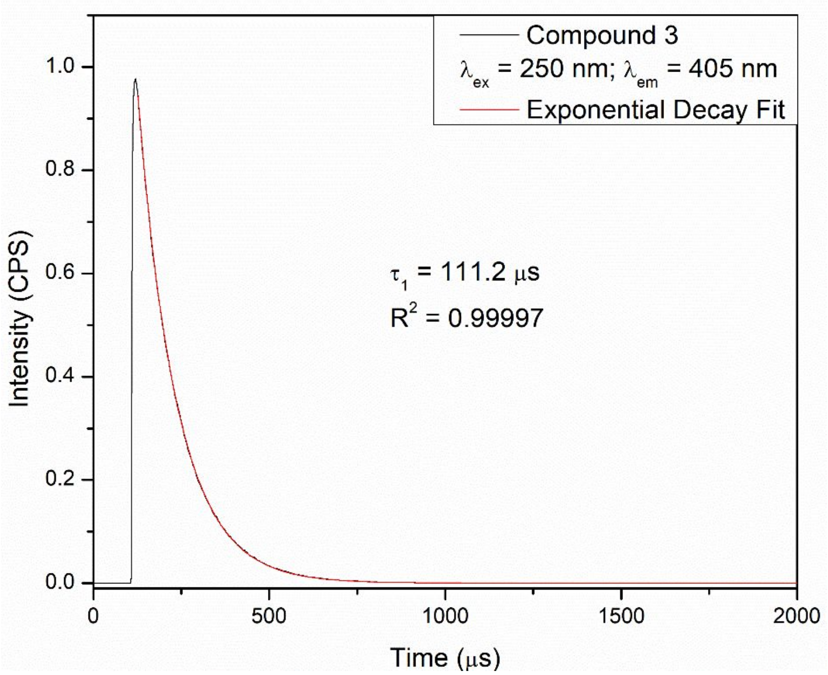

Figure S15. Experimental phosphorescence decay plot of **3** (black) fit with a single exponential decay function (red). The excitation wavelength was 250 nm and the emission wavelength was 405 nm.

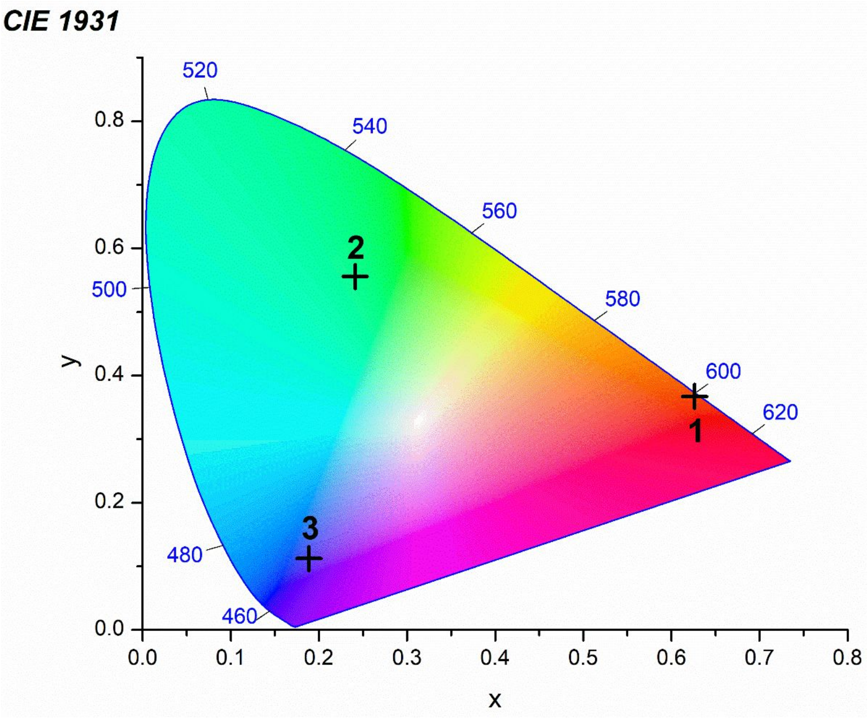

Figure S16. CIE 1931 plot for **1-3** highlighting the significant shifts in chromaticity dependent upon the identity of the arene ligand.

VI. Ligand Photoluminescence Spectra

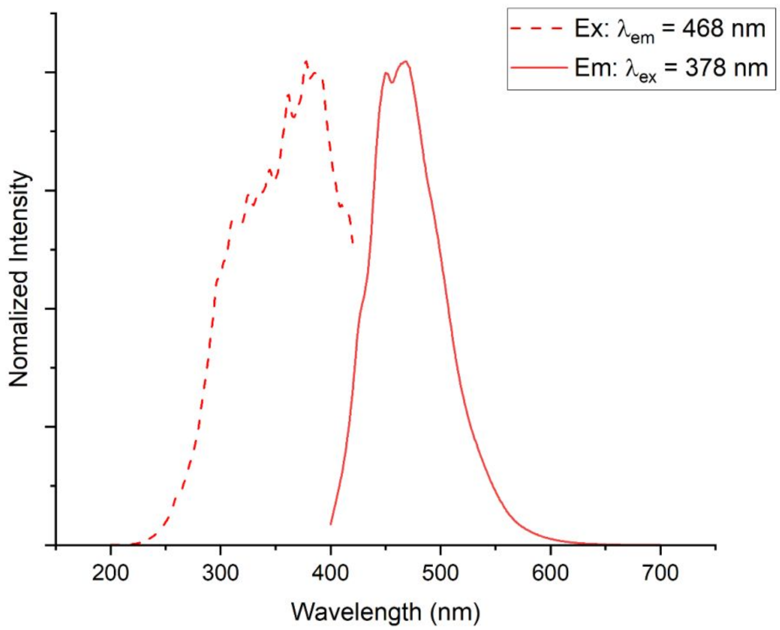

Figure S17. Solid-state excitation and emission spectra of pyrene.

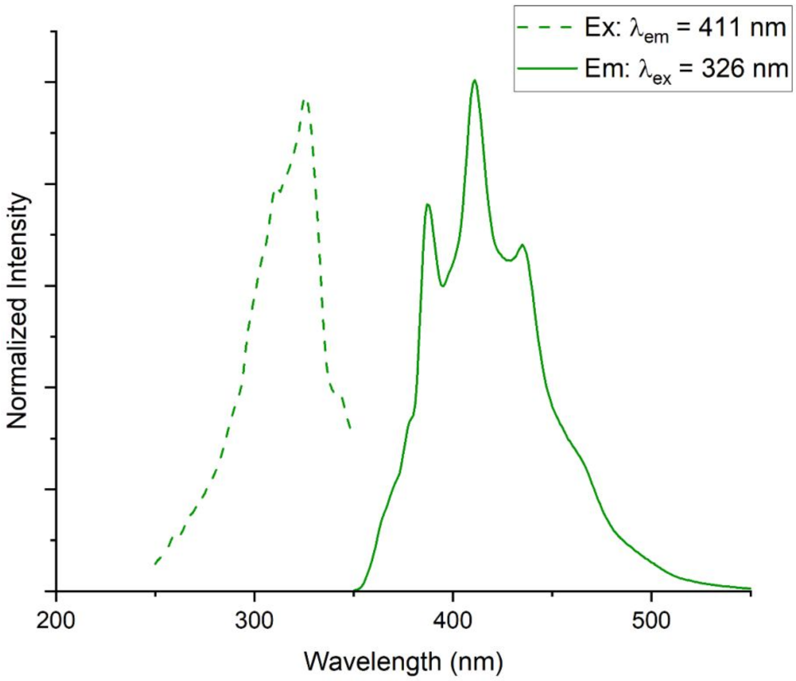

Figure S18. Solid-state excitation and emission spectra of naphthalene.

VII. Thermogravimetric Analysis

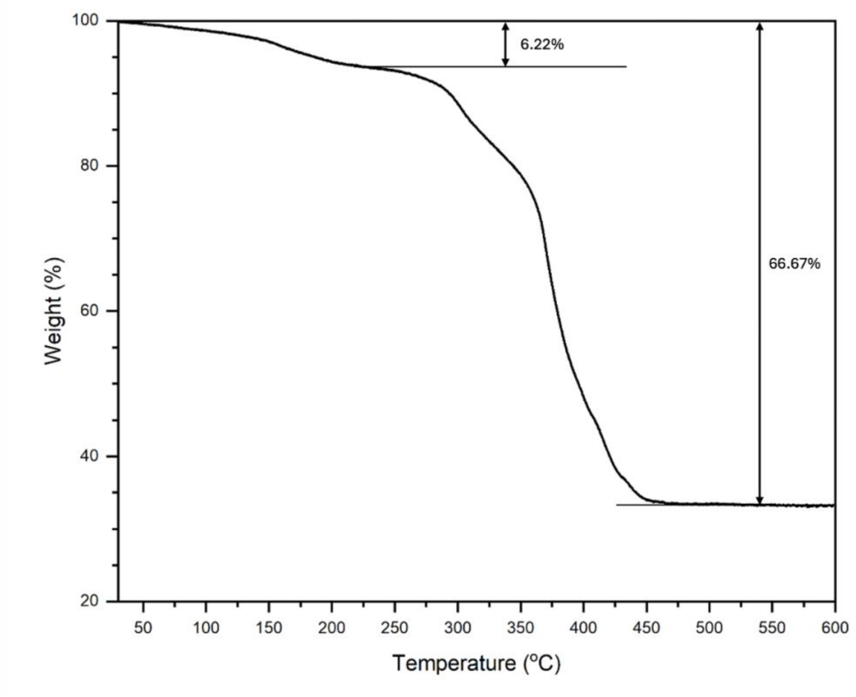

Figure S19. Thermogravimetric analysis plot for **1** collected over 30-600 °C.

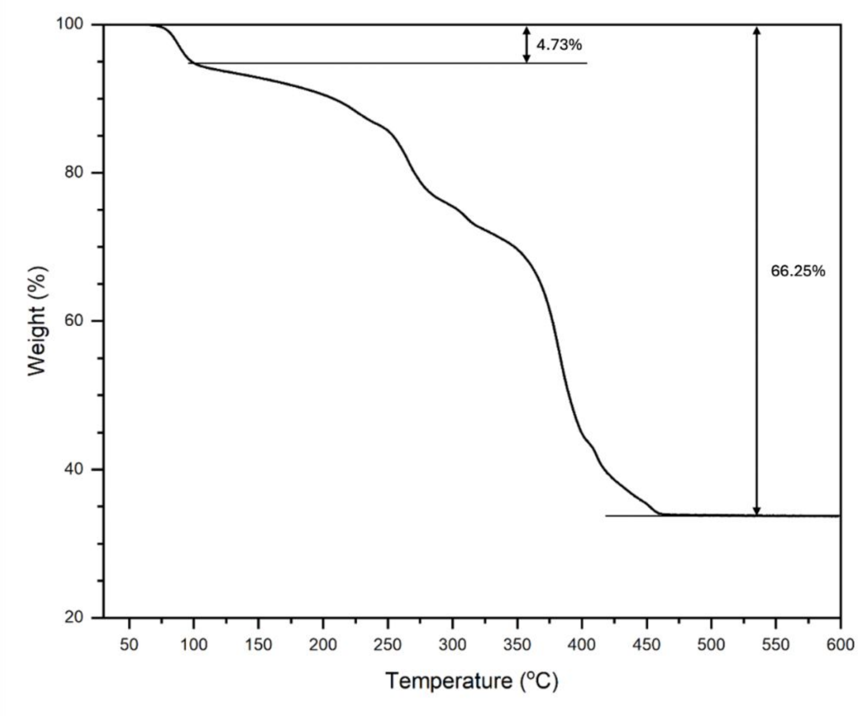

Figure S20. Thermogravimetric analysis plot for **2** collected over 30-600 °C.

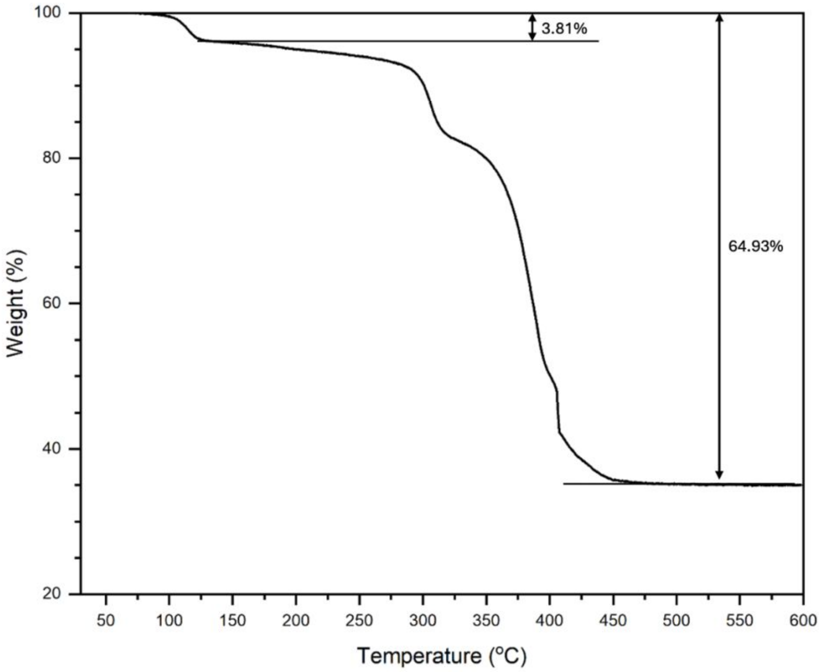

Figure S21. Thermogravimetric analysis plot for **3** collected over 30-600 °C.

VIII. Summary of Supramolecular Interactions

Table S1. Selected noncovalent interactions in compounds **1-3**. Values for reasonable D-H···A distances, LP-π distances, and centroid···centroid (Cg···Cg) distances for interactions were obtained from literature.[1-4] An asterisk (*) denotes an atom generated through symmetry.

Compound 1: **Cg(1)** = N11, C12, C13, C14, C15, C16; **Cg(3)** = C31, C32, C33, C34, C35, C36; **Cg(4)** = C31, C32, C31*, C36*, C37*, C38*; **Cg(5)** = C31, C36, C37, C38, C31*, C32*

Symmetry codes: i) -x+1, -y+2, -z; ii) x+1, y, z

Compound 2: Cg(1) = N11, C12, C13, C14, C15, C16; **Cg(3)** = C31, C32, C33, C33*, C34*, C35*; **Cg(4)** = C33, C34, C35, C31*, C32*, C33*

Symmetry codes: i) x, y-1, z; ii) -x, -y, -z

Compound 3: Cg(1) = N11, C12, C13, C14, C15, C16; **Cg(4)** = C31B, C32B, C33B, C34B, C35B

Symmetry codes: i) -x, -y, -z; ii) x-1, y, z

Compound	Interaction	D-H···A (Å)	D-H···A ∠ (°)	Cg···Cg or Bi ···Cg (Å)	Slip angle (°)
**1**	O(24)-H(24A) --- O(41)	2.493(5)	178(8)	-	-
	O(41)-H(41A) --- O(14)	2.743(5)	162(5)	-	-
	O(41)-H(41B) --- O(14)	2.722(4)	168(4)	-	-
	Cg(1) ··· Cg(3)	-	-	3.569(3)	19.3
	Cg(1) ··· Cg(4)	-	-	3.861(3)	29.2
	Cg(1) ··· Cg(5)	-	-	3.600(3)	20.8
	Bi ··· Cg(3)	-	-	3.889	22.2
**2**	O(24)-H(24A) --- O(41)	2.488(5)	168(3)	-	-
	O(41)-H(41A) --- O(13)	2.755(5)	168(6)	-	-
	O(41)-H(41B) --- O(14)	2.741(5)	164(5)	-	-
	Cg(1) ··· Cg(3)	-	-	3.556(4)	23.9
	Cg(1) ··· Cg(4)	-	-	3.555(4)	23.9
	Bi ··· Cg(3)	-	-	3.579	10.2
**3**	O(24)-H(24A) --- O(41)	2.482(5)	165(6)	-	-
	O(41)-H(41B) --- O(13)	2.754(4)	167(6)	-	-
	O(41)-H(41A) --- O(14)	2.766(5)	156(6)	-	-
	Cg(1) ··· Cg(4)	-	-	3.37(4)	14.8

IX. References

1. Janiak, C. A critical account on pi-pi stacking in metal complexes with aromatic nitrogen-containing ligands. *J. Chem. Soc., Dalton Trans.* **2000,** (21), 3885-3896.
2. Steiner, T. C-H-O Hydrogen Bonding in Crystals. *Crystallogr. Rev.* **1996,** *6*, 1-51.
3. Steiner, T. The Hydrogen Bond in the Solid State. *Angew. Chem. Int. Ed.* **2002,** *41*, 48-76.
4. Caracelli, I.; Haiduc, I.; Zukerman-Schpector, J.; Tiekink, E.R.T. Delocalised antimony(lone pair)- and bismuth(lone pair)-...π(arene) interactions: Supramolecular assembly and other considerations. *Coord. Chem. Rev.* **2013**, 257(11-12), 2863-2879.
